# Androgen deprivation therapy-related fracture risk in prostate cancer: an insurance claims database study in Japan

**DOI:** 10.1007/s00774-024-01497-4

**Published:** 2024-03-17

**Authors:** Hisashi Matsushima, Tetsuya Taguchi, Sho Kodama, Naoki Okubo, Kengo Saito, Katarzyna Jabłońska, Seiji Fukumoto, Toshio Matsumoto

**Affiliations:** 1 Department of Urology, Tokyo Metropolitan Police Hospital, 4-22-1 Nakano, Nakano-ku, Tokyo, 164-8541 Japan; 2 Division of Endocrine and Breast Surgery, Kyoto Prefectural University of Medicine, Kyoto, Japan; 3 Primary Medical Science Department, Medical Affairs Division, Japan Business Unit, Daiichi Sankyo Co., Ltd., Tokyo, Japan; 4 Data Intelligence Department, Global DX, Daiichi Sankyo Co., Ltd., Tokyo, Japan; 5 Putnam PHMR, Kraków, Poland; 6 Tokushima University, Tokushima, Japan

**Keywords:** Prostate cancer, Androgen-deprivation therapy, Osteoporosis, Epidemiology, Fracture risk, Japan

## Abstract

**Introduction:**

Androgen deprivation therapy (ADT) is widely used for the treatment of prostate cancer. ADT is associated with reduced bone density leading to an increased risk of osteoporotic fracture. The objective of this retrospective cohort study was to quantify fracture risk in men treated with ADT for prostate cancer in real-world practice in Japan.

**Materials and methods:**

Data were extracted from the Japanese Medical Data Vision (MDV) database. Men initiating ADT for treatment of prostate cancer between April 2010 and March 2021 were identified and matched to a cohort of prostate cancer patients not taking ADT using a propensity score. Fracture rates were estimated by a cumulative incidence function and compared between cohorts using a Cox cause-specific hazard model. Information was extracted on demographics, comorbidities and bone densitometry.

**Results:**

30,561 men with PC starting ADT were matched to 30,561 men with prostate cancer not treated with ADT. Following ADT initiation, <5% of men underwent bone densitometry. Prescription of ADT was associated with an increased fracture risk compared to not taking ADT (adjusted hazard ratio: 1.63 [95% CI 1.52–1.75]).

**Conclusion:**

ADT is associated with a 1.6-fold increase in the risk of osteoporotic fracture in men with prostate cancer. Densitometry in this population is infrequent and monitoring urgently needs to be improved in order to implement effective fracture prevention.

**Supplementary Information:**

The online version contains supplementary material available at 10.1007/s00774-024-01497-4.

## Introduction

Worldwide, prostate cancer (PC) is the second most prevalent type of cancer in men. In 2020, an estimated 1.4 million new cases and 375,304 PC-related deaths occurred worldwide [1]. In Japan, the age-standardised incidence rate for 2020 was 51.8 cases/100,000 and the age-standardised mortality rate was 4.5 deaths/100,000 [1]. Although incidence has risen in Japan over the last twenty years, PC-related mortality is declining [1], perhaps due to early detection and improved treatment. The aetiology of PC is multifactorial, with risk factors including older age, family history of PC, and African ancestry [2]. Nutritional, geographical, genetic and epigenetic factors are also important [3].

The development and progression of PC is, at least in part, driven by sex hormones (androgens) [4]. For this reason, lifelong androgen deprivation therapy (ADT), potentially in combination with anti-androgens has become the standard of care for treatment of men with advanced metastatic PC [4]. In addition, the National Comprehensive Cancer Network guidelines recommend the combination of radiotherapy and short or long-term ADT in high-risk non-metastatic PC [5]. However, use of ADT will also deprive other tissues whose integrity in men is also androgen-dependent of trophic support. In particular, androgens (and oestrogens in women) are important for maintaining bone homeostasis and, in their absence, bone resorption occurs more actively than bone formation, leading to a net decrease in bone mass [6]. Several mechanisms may contribute to this effect. Firstly, testosterone stimulates osteoblast proliferation and suppresses apoptosis, whilst at the same time inhibiting bone resorption by osteoclasts both directly and through suppressing secretion of RANKL which stimulates osteoclast-mediated bone resorption [7, 8]. If testosterone signalling is interrupted by ADT, then bone homeostasis will be perturbed in favour of net bone loss [7]. Cancer treatment-induced bone loss (CTIBL) is thus an important clinical issue in men receiving ADT for PC and in women treated with aromatase inhibitors for breast cancer [9]. Patients with PC who receive ADT with luteinising hormone releasing hormone (LH-RH) agonists or antagonists in combination with antiandrogens have been demonstrated to undergo a rapid decrease in bone mass [10, 11] and to be at increased risk of osteoporosis [12], osteoporotic fracture [13–16] and fracture-related mortality [17].

The International Osteoporosis Foundation (IOF) published a position paper on the prevention of fragility fractures in patients with PC in 2017 [18]. This recommended systematic risk assessment in patients starting hormonal therapy for non-metastatic PC and starting antiresorptive treatment in patients at high risk. In 2020, the Japanese Society for Bone and Mineral Research (JSBMR) issued a manual for the treatment of CTIBL [19]. According to this manual, patients starting ADT for PC should undergo a risk assessment including bone mineral density measurement, documentation of a family history of hip fracture and the FRAX® Fracture Risk Assessment Tool [20]. Based on this evaluation, a decision should be made about initiating prophylaxis with antiresorptive medications to prevent bone loss.

The only source of data on CTIBL in Japan comes from a limited number of randomised controlled trials of anti-osteoporotic drugs and there are no epidemiological data or data on fracture risk evaluated on a large scale in the real-world setting. In order to address this knowledge gap, health insurance databases represent a powerful resource for acquiring such information. The objective of this study is to estimate fracture risk in patients with PC treated with ADT in Japan using information from a health insurance claims database covering acute-care hospitals distributed throughout Japan.

## Materials and methods

This was a retrospective cohort study of fracture outcome in men receiving ADT for the treatment of PC, performed using data from the Medical Data Vision (MDV) health insurance claims database from Japan. The study period lasted fourteen years, from 1st April 2009 and 31st March 2022. All patients with a confirmed diagnosis of PC during this study period were eligible. The study is registered in the University hospital Medical Information Network Clinical Trials Registry under the identifier UMIN000048942 (date of registration, September 15, 2022).

Eligible patients were divided into two cohorts based on whether or not they received hormone therapy (ADT+ and ADT− cohorts), and these two cohorts were matched using a propensity score. For the purposes of this study, hormone therapy was defined as at least one prescription during the selection period (from 1st April 2010 to 31st March 2021) for one of the following: degarelix acetate, goserelin acetate, leuprorelin acetate, bicalutamide, flutamide, apalutamide, darolutamide, enzalutamide and abiraterone acetate.

The index diagnosis date was defined as the first retrieved diagnosis of PC during the selection period. In the ADT+ cohort, the treatment initiation date was defined as the first retrieved claim for ADT prescription following the index diagnosis date. In the ADT− cohort, a dummy treatment initiation date was defined as the index diagnosis date plus the number of days between the index diagnosis date and the treatment initiation date of the matched ADT+ case (after performing propensity score matching). The baseline period was defined as the period of at least twelve months preceding the index diagnosis date. The follow-up period was defined as the period lasting from the treatment initiation date until 31st March 2022, or until the patient died or left the database. The study design is illustrated in Supplementary Fig. 1.

### Data source

Data was extracted from the MDV insurance claims database [21]. This database includes information on all reimbursement claims relating to healthcare delivered in participating hospitals implementing the Japanese Diagnosis Procedure Combination (DPC) fixed-payment reimbursement system. Over 400 acute-care hospitals currently participate in the MDV, accounting for > 20% of such hospitals in Japan and > 36 million patients of all ages have been included since the database became available in 2008. Information is anonymised through a double encryption system.

Claims relating to both inpatient and outpatient healthcare are documented in the database, including hospital stays, physician consultations, procedures and tests, and medication delivery. Reasons for hospitalization are identified by a diagnostic code based on the International Classification of Diseases, 10th Edition (ICD-10), together with a specific identifier related to the Japanese vernacular name. Demographic data are limited to age and gender. Information is also available on the hospital facility and department in which the patient was hospitalised. Mortality information is limited to in-hospital deaths. However, clinical data, such as the results of tests or the reason for medication prescription are not systematically available. In addition, patient identifiers are hospital-specific so individuals cannot be tracked across hospitals if they consult at different facilities.

### Subjects

All patients aged 18 years or older at the index diagnosis date with a confirmed diagnosis of PC based on the relevant ICD-10 codes and identifiers were eligible (Supplementary Table 1). Patients were excluded if they had a baseline period lasting less than twelve months prior to the index diagnosis date or if they had no documented claim in the database during the twelve months following the treatment initiation date. The following groups of patients were also excluded: (i) men exposed to hormone therapy during the baseline period; (ii) men with any claim during the study period related to another disease potentially resulting in reduced bone density (see list in Supplementary Table 1); (iii) men with at least two claims with a disease code for osteoporosis and at least one prescription of a specific osteoporosis drug during the baseline period, and (iv) men with at least one claim with a disease code for fracture during the baseline period.

### Study variables

Study variables of interest included demographic variables, treatment-related variables, fracture-related variables, comorbidities of interest and mortality. Demographic variables were limited to age and gender at the index diagnosis date. Treatment variables were the prescription of an LH-RH agonist, LH-RH antagonist or an antiandrogen at least once during the follow-up period. These treatments were LH-RH agonists or antagonists (degarelix acetate, goserelin acetate or leuprorelin acetate) and antiandrogens (abiraterone acetate, apalutamide, bicalutamide, darolutamide, enzalutamide or flutamide). Treatments were identified from the relevant Anatomical Therapeutic Chemical (ATC) code in prescription reimbursement claims. Fragility fractures occurring during the follow-up period were identified from ICD-10 codes and the associated Japanese vernacular name. Fractures of interest were fractures of the vertebra, sternum, rib, pelvis, clavicle, scapula, humerus, forearm, femur and lower leg. Claims for bone mineral density testing were retrieved. Comorbidities of interest were dementia, diabetes, chronic kidney disease, sleep disorders, alcoholism and rheumatoid arthritis. These were identified from the appropriate ICD-10 codes and associated Japanese vernacular names associated with the reimbursement claim. With regard to mortality, only information on in-hospital deaths was available; the date of death was documented. Finally, the size of the care facility where care was delivered (assigned to one of three bed size categories: ≤ 199 beds, 200 to 499 beds and ≥ 500 beds) and the medical speciality of the department were documented.

### Outcomes

The principal outcome of interest was the cumulative incidence of all fractures, vertebral fractures, non-vertebral fractures and hip fractures following the treatment initiation date. The proportion of patients undergoing bone mineral density testing in the six months preceding and the six months following the treatment initiation date was also evaluated in each cohort.

### Statistical analysis

#### Propensity score matching

In order to ensure comparability of subjects between the different cohorts and to minimise confounding by covariates, a propensity score was calculated and used to match the ADT+ and ADT− cohorts pairwise [22–24]. The propensity score was computed using logistic regression, with study cohort as the dichotomous dependant variable. The independent variables considered were age at the index diagnosis date, the calendar year at the index diagnosis date, the number of beds in the hospital where the reimbursement claim was issued, the hospital department where the most medical resources was used and comorbidities documented during the baseline period.

The resulting propensity score was then determined individually for each subject in each cohort. For each patient in the ADT+ cohort, a patient in the ADT− cohort was matched. Matching was performed 1:1 by the propensity score on a nearest neighbour basis using a pre-defined calliper, initially set at 0.25 times the standard deviation of the logit-transformed propensity score. The quality of matching was evaluated from the standard mean difference (SMD) and variance ratio (VR) between the matched pairs.

#### Descriptive statistics

Patient characteristics were compared pairwise between the matched ADT+ and ADT− cohorts. Continuous variables are presented as mean values with standard deviations (SD) or median values with interquartile range (IQR), as appropriate.

#### Fracture incidence and time-to-event analysis

Time-to-event analysis was performed to compare fracture incidence between cohorts over time, with death as a competing risk, using a cumulative incidence function [25]. The crude fracture incidence and the fracture incidence rate were calculated with their 95% confidence interval every year after the treatment initiation date. Any difference in time to fracture between the matched ADT+ and ADT− cohorts was evaluated using a Cox cause-specific hazard model, adjusted for potential confounders [26]. These confounders were age at the treatment initiation date and the presence of comorbidities during the 12 months preceding the treatment initiation date (pre-treatment).

### Ethics

The study complied with all relevant international and national legislation on medical research and data privacy. In particular, it complied with the Declaration of Helsinki and with the Japanese Act on the Protection of Personal Information. This research was approved by an ethical review committee, Research Institute of Healthcare Data Science from an ethical and scientific perspective under the Ethical Guidelines for Medical Research Involving Human Subjects (partially revised on 6th June 2022) was used as a reference.

## Results

### Study population

**Fig. 1:**
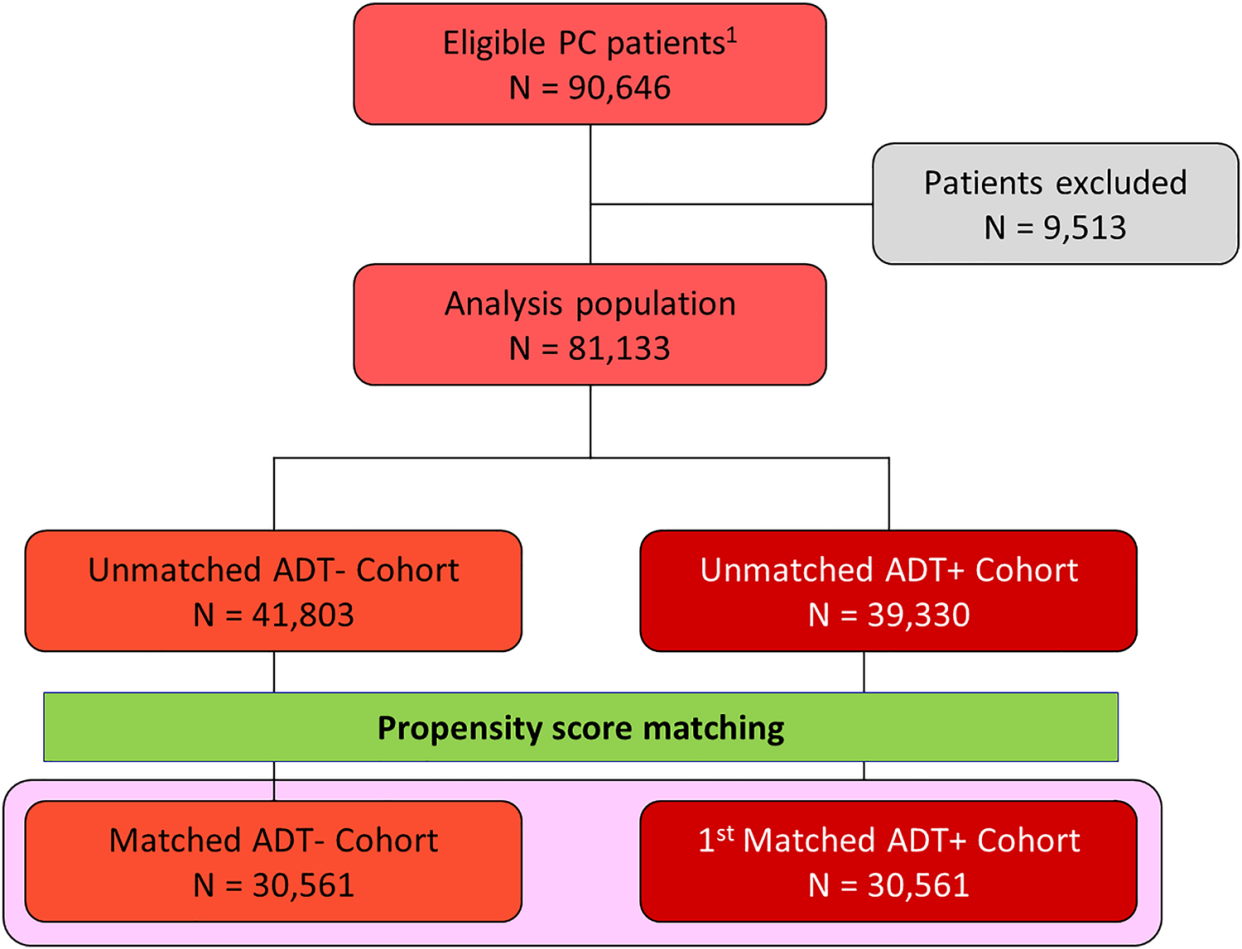
Patient selection flowchart. ADT+: patients under hormone therapy; ADT−: patients not taking hormone therapy; PC: prostate cancer. ^1^Patients with a confirmed diagnosis of PC during the study period (1st April 2009 to 31st March 2022) and with at least 12 months baseline period and at least one claim registered in the 12 months after the treatment initiation date

During the selection period, 90,646 patients with PC aged ≥ 18 years with a baseline period and a follow-up period of at least 12 months were identified in the MDV database. Of these, 9513 were excluded, principally because they were exposed to hormone therapy during the baseline period, because they had claims related to osteoporosis or osteoporotic fractures (as defined in the “Methods”), were documented prior to the index diagnosis date or because they had claims related to another disease potentially resulting in reduced bone density documented at least once during the follow-up period. The remaining patients corresponded to the analysis population and consisted of 39,330 patients under hormone therapy and 41,803 not receiving hormone therapy. After propensity score matching, the two matched cohorts consisted of 30,561 patients each (Fig. 1). For the matched ADT+ cohort and ADT− matched pair, the median follow-up duration was 2.75 years [IQR: 1.50–4.56] in the ADT+ cohort and 2.30 years [1.03–4.23] in the ADT− cohort.

The characteristics of the matched populations and the quality of matching are presented in Table 1. The quality of matching between the ADT+ and ADT− cohorts was good, with an SMD < 0.1 for all variables except age (0.18). For the ADT+ and ADT− matched cohorts the median age was 75 years and 73 years respectively.

**Table 1:** Quality of propensity score matching of the ADT + and ADT- cohorts

	ADT+ cohort N = 30,561	ADT− cohort N = 30,561	SMD	VR
Age at index diagnosis date^a^ [years]			0.18	0.94
Mean ± SD	75.6 ± 7.7	74.2 ± 7.9		
Median [Q1; Q3]	75.0 [70.0; 81.0]	73.0 [69.0; 79.0]		
Calendar year at index diagnosis date			0.03	0.88
Mean ± SD	2017.3 ± 2.5	2017.4 ± 2.6		
Median [Q1; Q3]	2018 [2016; 2019]	2018 [2016; 2019]		
Medical facility category				
199 or less	2,309 (7.6%)	2,138 (7.0%)	0.02	1.07
200 to 499	18,564 (60.7%)	17,510 (57.3%)	0.07	0.97
500 or more	9688 (31.7%)	10,913 (35.7%)	0.08	0.94
Medical department				
Urology	27,144 (88.8%)	26,521 (86.8%)	0.05	0.87
Internal medicine	4179 (13.7%)	3678 (12.0%)	0.05	1.12
Orthopaedics	1918 (6.3%)	1284 (4.2%)	0.10	1.46
General surgery	1532 (5.0%)	1236 (4.0%)	0.04	1.23
Radiology	1451 (4.7%)	1317 (4.3%)	0.02	1.10
Cardiology	1033 (3.4%)	864 (2.8%)	0.03	1.19
Gastroenterological internal medicine	969 (3.2%)	846 (2.8%)	0.02	1.14
Neurosurgery	793 (2.6%)	607 (2.0%)	0.04	1.30
Gastrointestinal medicine	765 (2.5%)	639 (2.1%)	0.03	1.19
Rehabilitation	369 (1.2%)	289 (0.9%)	0.03	1.27
Comorbidities in the baseline period^b^				
Dementia	968 (3.2%)	718 (2.3%)	0.05	1.34
Diabetes	8,468 (27.7%)	7,028 (23.0%)	0.11	1.13
Chronic kidney disease	1,448 (4.7%)	1,128 (3.7%)	0.05	1.27
Sleep disorder	4,320 (14.1%)	3,361 (11.0%)	0.10	1.24
Alcoholism	33 (0.1%)	29 (0.1%)	0.00	1.14
Rheumatoid arthritis	358 (1.2%)	264 (0.9%)	0.03	1.35

*IQR* interquartile range, *SD* standard deviation, *SMD* standard mean difference, *VR* variance ratio

^a^The index diagnosed date was the date of the first claim during the selection period for the NC cohort

^b^Multiple responses were possible

### Hormone treatment

In the matched ADT+ cohort, patients were assigned to the three prespecified ADT treatment groups. Since they could receive more than one type of treatment (either simultaneously or sequentially), these groups are not mutually exclusive. In this way, 28,707 patients (93.9%) were prescribed an LH-RH agonist or antagonist at least once, 26,348 (86.2%) an anti-androgen. For oral treatments, the median duration of prescription was 376 days [IQR: 112.0–780.0] days. For injectable treatments, the mean number of injections prescribed was 7 [IQR: 4.0–13.0].

### Bone mineral density testing

Prior to the treatment initiation date, the proportion of men having undergone bone mineral density testing was very low (< 1.0%) (Table 2). In the ADT+ cohort, this proportion increased slightly in the 6 months following the treatment initiation to 2.6%.

**Table 2:** Bone mineral density testing

	ADT+ and ADT-− matched cohorts	
	ADT+ (N = 30,561)	ADT− (N = 30, 561)
BMD test in the 6 months before the treatment initiation date	239 (0.8%)	123 (0.4%)
BMD test in the 6 months following the treatment initiation date	785 (2.6%)	134 (0.4%)

### Fracture incidence

**Fig. 2:**
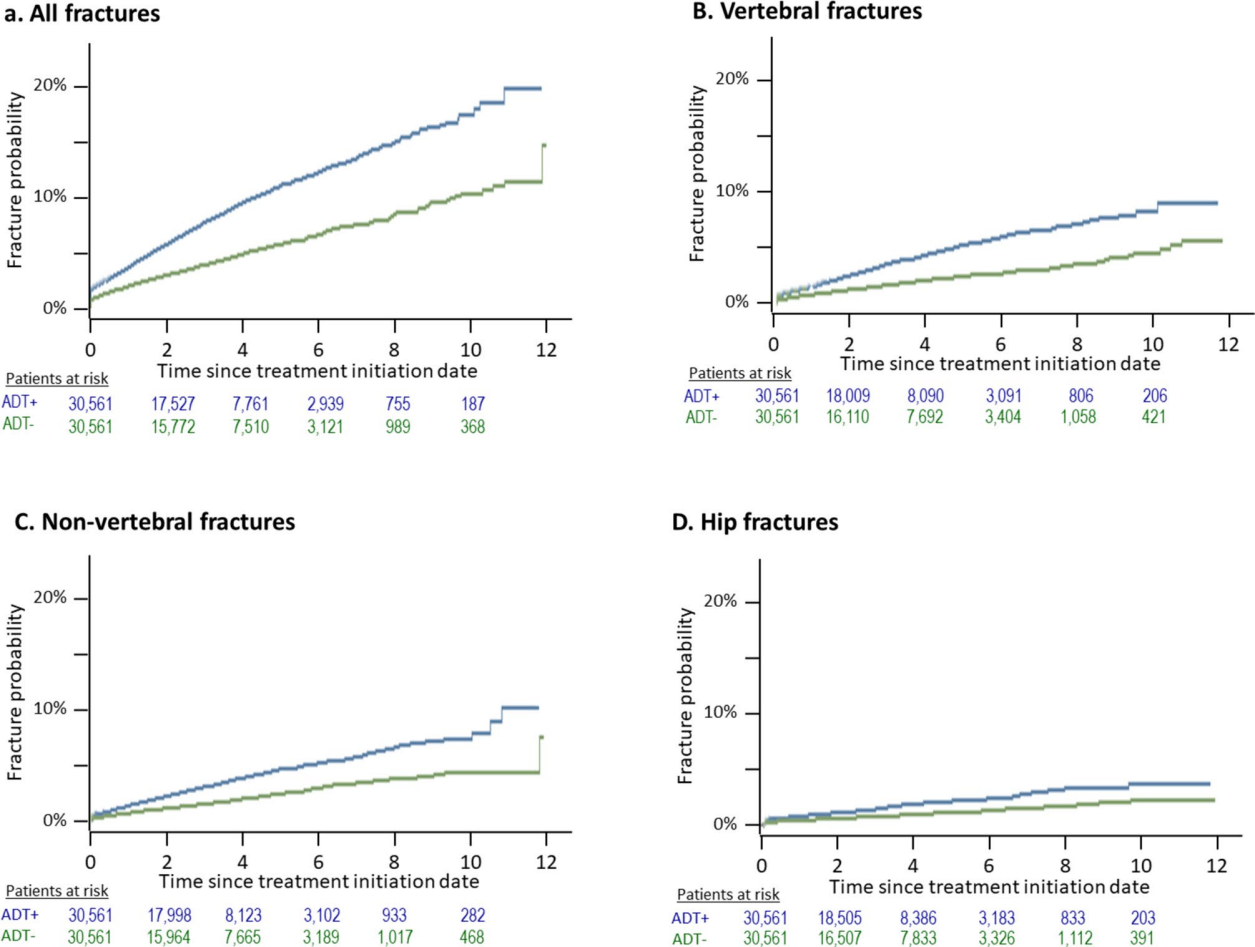
Fracture incidence rates in patients with PC as a function of hormone treatment. Data are presented as cumulative incidence functions with death as a competing risk. ADT+ blue curves): patients with PC under hormone therapy; ADT− (green curves): patients with PC not taking hormone therapy.

The cumulative incidence function curve for the four fracture types of interest in the two cohorts are presented in Fig. 2.

The crude cumulative fracture incidence at five years in the ADT+ cohort was 0.11 [95% CI 0.11–0.12] for all fractures, 0.02 [95% CI 0.02–0.02] for hip fractures, 0.05 [95% CI 0.05–0.05] for vertebral fractures and 0.05 [95% CI 0.04–0.05] for non-vertebral fractures. The crude cumulative fracture incidence at five years in the ADT− cohort was 0.06 [95% CI 0.05–0.06] for all fractures, 0.01 [95% CI 0.01–0.01] for hip fractures, 0.02 [95% CI 0.02–0.03] for vertebral fractures and 0.03 [95% CI 0.02–0.03] for non-vertebral fractures. Crude cumulative fracture incidence rates at each year for all fracture sites are presented in Supplementary Table 2.

**Fig. 3:**
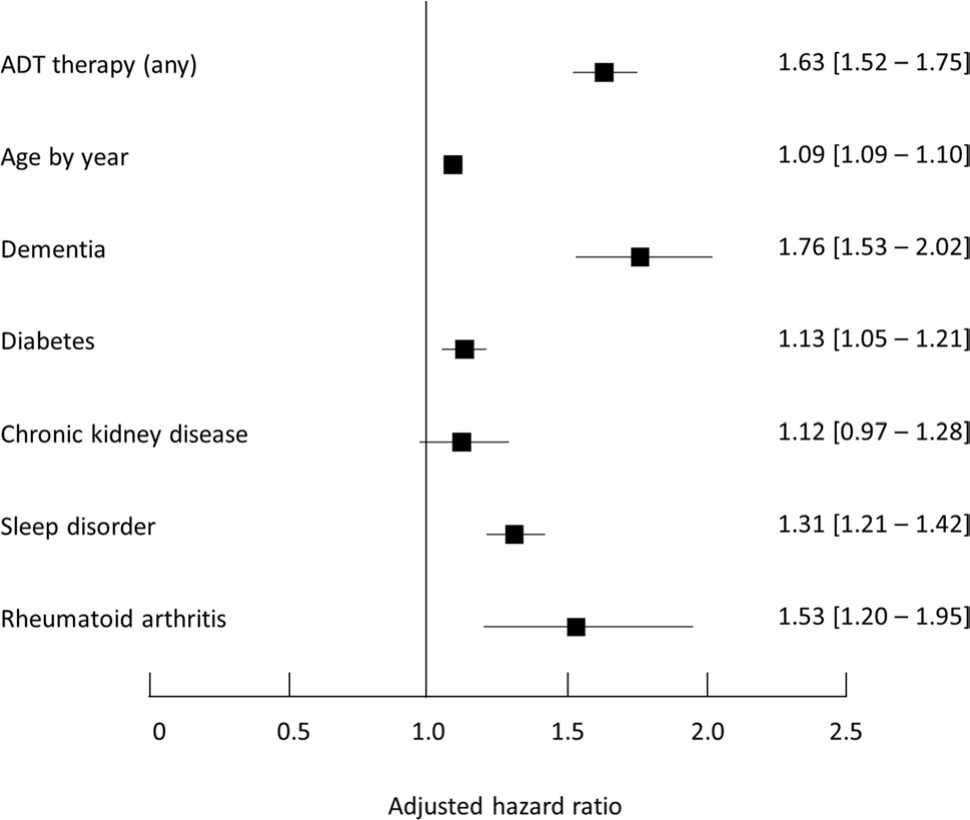
Cause-specific Cox hazard model for fracture risk (all fractures). Data are presented in the form of a Forest plot showing as hazard ratios with their 95% confidence intervals. For each categorical variable, the reference category was ‘absent’. Age was included in the model as a continuous variable and the hazard ratio presented by year. ADT+: patients with PC under hormone therapy; ADT−: patients with PC not taking hormone therapy; AA: anti-androgen; LH-RH agent: luteinising hormone releasing hormone agonist or antagonist

In the Cox cause-specific hazard model comparing the matched ADT+ and ADT− cohorts for all fractures combined, use of hormone therapy was associated with an increased fracture risk (*p* < 0.001), with an adjusted hazard ratio of 1.63 [95% CI 1.52–1.75] (Fig. 3). This incremental risk was of similar magnitude to that conferred by dementia (HR: 1.76 [1.53–2.02]) and rheumatoid arthritis (HR: 1.53 [1.20–1.95]) (Fig. 3). The increased risk attributable to hormone therapy was observed for all individual fracture sites evaluated and was of similar magnitude: HR: 1.75 [1.57–1.96] for vertebral fractures, 1.65 [1.48–1.84] for non-vertebral fractures and 1.55 [1.32–1.82] for hip fractures. Full information on the results of the Cox cause-specific hazard model for all fracture sites is provided in Supplementary Table 3. In all cases the risk associated with ADT was independent of the other risk factors tested in the Cox cause-specific hazard model, such as age and comorbidities.

## Discussion

This study in a large Japanese health insurance claims database has demonstrated that men receiving hormone therapy for the treatment of PC are at increased risk for osteoporotic fracture. This increased risk was apparent from the start of treatment and was statistically significant from 1 year, the first time-point measured. An increased risk of fracture in men receiving hormone therapy was observed for all three sites of fracture evaluated (vertebral, non-vertebral and hip fractures).

The hazard ratio for fracture associated with hormone therapy for PC was 1.63 [95% CI 1.52–1.75] compared to untreated patients. This hazard ratio was of a similar magnitude to that observed for dementia (1.76 [1.53–2.02]) and for rheumatoid arthritis (1.53 [1.20–1.95]) in the same population, which are both established major risk factors for osteoporotic fracture [27, 28].

The hazard ratio reported in the present study is higher than that reported in a meta-analysis of studies of fracture risk in men receiving ADT performed prior to May 2008 in Europe and North America [29], which was 1.23 ([95% CI 1.10–1.38]. This difference may be explained by the fact that at the time of the meta-analysis, second generation antiandrogens were not available. On the basis of an insurance claims database study in Taiwan, Wu et al. suggested that the impact of ADT on osteoporotic fracture risk may be lower in East Asian that in European populations [15], but the current findings in a Japanese population are not consistent with this hypothesis.

Practice guidelines recommend bone densitometry in all men starting ADT for PC [18, 19]. The guidelines published by the IOF [18] and by the JSBMR [19] recommend monitoring of bone density in men at low fracture risk every 18 to 24 months and, in men at high risk, annually, along with upfront antiresorptive treatment. However, this is clearly not happening in everyday practice in Japan, where < 3% of men in our ADT+ cohort starting ADT were tested in the six months following the treatment initiation date. However, these monitoring rates may be underestimated if densitometry was performed at a different care facility to that at which ADT was prescribed. We have previously reported a significant treatment gap in bone density monitoring in another group of patients at risk for osteoporotic fractures, namely those treated with glucocorticoids, of whom only 6.5% were monitored [30]. However, in the case of men starting ADT, the gap is even bigger. The findings suggest a rupture in the continuity of care between the urologist responsible for managing PC and for prescribing hormone therapy and the orthopaedic surgeon responsible for fracture risk assessment and for prescribing antiresorptive treatment. Possible reasons for the low frequency of densitometry include not all hospitals being equipped for it, and importantly, that men in general are perceived as being at low risk for osteoporosis [31]. This erroneous perception may be due to urologists treating PC being not particularly interested in CTIBL and being poorly informed about it. Our findings emphasise the importance of educating urologists about the clinical importance of CTIBL.

A multidisciplinary osteoporosis liaison service for bone fracture prevention was initiated by the Japan Osteoporosis Society in 2012 [32], and urologists treating men with PC perhaps need to be encouraged to make use of this service. Further research will be required to evaluate why the use of densitometry is so low, in spite of it being recommended in practice guidelines.

In this study, we used a propensity score to take into account potential confounding factors and this enabled a high-quality match between the ADT+ and ADT− cohorts, and for this reason, the association between hormone therapy use and fracture risk can be considered robust. However, there are a number of known risk factors for osteoporotic fracture are not documented in the source database, such as smoking status and low body weight, and some residual confounding by such factors cannot be excluded.

In order to avoid ambiguities in the coding of the type of fracture, we included all fractures. For this reason, some of the events documented may have been high-energy traumatic rather than fragility fractures. It was thus impossible to document precisely the number of patients with high-energy fractures in this study. In a study evaluating the contribution of high-energy fractures to total fractures performed in Wales [33], it was observed that high energy fractures accounted for 12% of total fractures in individuals aged ≥ 60 years and 6% in individuals aged ≥ 80 years. For hip fractures, these proportions were 5% and 4% respectively. A study in Singapore reported that 7.3% of hip fractures in individuals aged ≥ 60 years were due to severe trauma [34]. Given that the mean age of our cohort was 75 years and that 27% were aged over eighty, we would estimate that < 10% of the fractures documented were high-energy fractures.

The strengths of the study include the large number of patients with PC available in the MDV database included (> 25,000 patients), which enabled the incidence of relatively rare fracture events to be estimated with precision, the availability of data on other medical potential confounding factors, and the use of a standardised coding system for healthcare resource use. There are also several limitations. Healthcare resource use claims are available only for hospitals participating in the MDV health insurance regimen and patients cannot be tracked across care facilities. This means that care delivered in hospitals other than the facility where the claim related to the index stay was issued will not be documented. In addition, since the claims only arise from acute-care hospitals qualified by the DPC reimbursement system, patients included in the study might not be strictly representative of the general Japanese population of men with PC. Moreover, use of other medications that may influence fracture risk, such as steroids or antiresorptive drugs has not been evaluated or taken into account. In addition, reasons for medication prescription are not documented; it was assumed that hormone therapies were prescribed for PC, but it cannot be excluded that they were used for other reasons. Mortality may be underestimated, since only deaths occurring in participating hospitals are documented. Underestimation of mortality will lead to an underestimate of the true fracture incidence as determined with the cumulative incidence function.

In conclusion, this real-world study provided information which will help increase awareness of CTIBL, and will bring knowledge that may help inform public health policies aimed at improving the prevention, diagnosis and treatment of CTIBL. In particular, systematic monitoring of bone density should be offered to all patients starting ADT for PC. This effort is necessary to reduce the incidence of fragility fractures, and thus improve the quality of life of patients with PC and reduce costs to the healthcare system.

### Supplementary Information

Below is the link to the electronic supplementary material.

Supplementary file1 (DOCX 71 KB)
