# Supplementary material for: Androgen deprivation therapy-related fracture risk in prostate cancer: an insurance claims database study in Japan
Source: J Bone Miner Metab. 2024 Mar 17;42(2):223–32. doi: 10.1007/s00774-024-01497-4 (PMC10982088; doi:10.1007/s00774-024-01497-4)
Supplement: Supplementary file 1 — Supplementary file1 (DOCX 71 KB) [file 774_2024_1497_MOESM1_ESM.docx]

**Androgen Deprivation Therapy-Related Fracture Risk in Prostate Cancer: an Insurance Claims Database Study in Japan**

**SUPPLEMENTARY MATERIAL**

## Supplementary Table 1.

## Disease codes used to identify prostate cancer.

| **ICD-10 code** | **ICD-10 name** | **Japanese disease code** | **Japanese disease code name** |
| --- | --- | --- | --- |
| C61 | Malignant neoplasm of prostate | 1859003 | Prostate cancer |
| C61 | Malignant neoplasm of prostate | 8848040 | Castration-resistant prostate cancer |
| C61 | Malignant neoplasm of prostate | 8848043 | Localized prostate cancer |
| C61 | Malignant neoplasm of prostate | 8848066 | Progressive prostate cancer |
| C61 | Malignant neoplasm of prostate | 8848074 | Prostate cancer recurrence |
| C795 | Secondary malignant neoplasm of bone and bone marrow | 8842788 | Bone metastases of prostate cancer |
| C798 | Secondary malignant neoplasm of other specified sites | 8848159 | Metastatic prostatic tumor |
| Z988 | Other specified postsurgical states | 8848075 | Postoperative status of prostate cancer |

## Disease codes used to identify diseases potentially reducing bone density

| **ICD-10 code** | **ICD-10 name** |
| --- | --- |
| E050 | Thyrotoxicosis with diffuse goitre |
| E051 | Thyrotoxicosis with toxic single thyroid nodule |
| E052 | Thyrotoxicosis with toxic multinodular goitre |
| E053 | Thyrotoxicosis from ectopic thyroid tissue |
| E055 | Thyroid crisis or storm |
| E058 | Other thyrotoxicosis |
| E059 | Thyrotoxicosis, unspecified |
| E210 | Primary hyperparathyroidism |
| E211 | Secondary hyperparathyroidism, not elsewhere classified |
| E213 | Hyperparathyroidism, unspecified |
| E214 | Other specified disorders of parathyroid gland |
| E215 | Disorder of parathyroid gland, unspecified |
| E291 | Testicular hypofunction |
| E283 | Primary ovarian failure |
| E240 | Pituitary-dependent Cushing disease |
| E241 | Nelson syndrome |
| E242 | Drug-induced Cushing syndrome |
| E243 | Ectopic ACTH syndrome |
| E244 | Alcohol-induced pseudo-Cushing syndrome |
| E248 | Other Cushing syndrome |
| E249 | Cushing syndrome, unspecified |
| E274 | Other and unspecified adrenocortical insufficiency |
| A099 | Gastroenteritis and colitis of unspecified origin |
| B181 | Chronic viral hepatitis B without delta-agent |
| B182 | Chronic viral hepatitis C |
| C900 | Multiple myeloma |
| D472 | Monoclonal gammopathy of undetermined significance (MGUS) |
| K529 | Noninfective gastroenteritis and colitis, unspecified |
| K703 | Alcoholic cirrhosis of liver |
| K743 | Primary biliary cirrhosis |
| K746 | Other and unspecified cirrhosis of liver |
| M804 | Drug-induced osteoporosis with pathological fracture |
| M8045 | Drug-induced osteoporosis with pathological fracture |
| M8046 | Drug-induced osteoporosis with pathological fracture |
| M8048 | Drug-induced osteoporosis with pathological fracture;Other |
| M8149 | Drug-induced osteoporosis;Site unspecified |
| N258 | Other disorders resulting from impaired renal tubular function |
| Q822 | Mastocytosis |

## Supplementary Table 2. Crude cumulative fracture incidence rates by year and by fracture site.

| **Year of follow-up** | **All fracture sites** | | **Vertebral fracture** | | **Non-vertebral fracture** | | **Hip fracture** | |
| --- | --- | --- | --- | --- | --- | --- | --- | --- |
|  | **ADT+** | **ADT-** | **ADT+** | **ADT-** | **ADT+** | **ADT-** | **ADT+** | **ADT-** |
| **1** | 0.04 [0.04 - 0.04] | 0.02 [0.02 - 0.02] | 0.02 [0.01 - 0.02] | 0.01 [0.01 - 0.01] | 0.02 [0.01 - 0.02] | 0.01 [0.01 - 0.01] | 0.01 [0.01 - 0.01] | 0.00 [0.00 - 0.00] |
| **2** | 0.06 [0.06 - 0.06] | 0.03 [0.03 - 0.03] | 0.02 [0.02 - 0.03] | 0.01 [0.01 - 0.01] | 0.02 [0.02 - 0.03] | 0.01 [0.01 - 0.01] | 0.01 [0.01 - 0.01] | 0.01 [0.00 - 0.01] |
| **3** | 0.08 [0.08 - 0.08] | 0.04 [0.04 - 0.04] | 0.04 [0.03 - 0.04] | 0.02 [0.01 - 0.02] | 0.03 [0.03 - 0.03] | 0.02 [0.01 - 0.02] | 0.01 [0.01 - 0.02] | 0.01 [0.01 - 0.01] |
| **4** | 0.10 [0.09 - 0.10] | 0.05 [0.05 - 0.05] | 0.04 [0.04 - 0.05] | 0.02 [0.02 - 0.02] | 0.04 [0.04 - 0.04] | 0.02 [0.02 - 0.02] | 0.02 [0.02 - 0.02] | 0.01 [0.01 - 0.01] |
| **5** | 0.11 [0.11 - 0.12] | 0.06 [0.05 - 0.06] | 0.05 [0.05 - 0.05] | 0.02 [0.02 - 0.03] | 0.05 [0.04 - 0.05] | 0.03 [0.02 - 0.03] | 0.02 [0.02 - 0.02] | 0.01 [0.01 - 0.01] |
| **6** | 0.12 [0.12 - 0.13] | 0.07 [0.06 - 0.07] | 0.06 [0.05 - 0.06] | 0.03 [0.02 - 0.03] | 0.05 [0.05 - 0.06] | 0.03 [0.03 - 0.03] | 0.02 [0.02 - 0.03] | 0.01 [0.01 - 0.01] |
| **7** | 0.14 [0.13 - 0.14] | 0.08 [0.07 - 0.08] | 0.06 [0.06 - 0.07] | 0.03 [0.03 - 0.03] | 0.06 [0.06 - 0.07] | 0.04 [0.03 - 0.04] | 0.03 [0.02 - 0.03] | 0.01 [0.01 - 0.02] |
| **8** | 0.15 [0.14 - 0.16] | 0.09 [0.08 - 0.09] | 0.07 [0.06 - 0.08] | 0.03 [0.03 - 0.04] | 0.07 [0.06 - 0.07] | 0.04 [0.04 - 0.05] | 0.03 [0.03 - 0.04] | 0.02 [0.01 - 0.02] |
| **9** | 0.16 [0.15 - 0.17] | 0.10 [0.09 - 0.11] | 0.07 [0.07 - 0.08] | 0.04 [0.03 - 0.05] | 0.07 [0.07 - 0.08] | 0.04 [0.04 - 0.05] | 0.03 [0.03 - 0.04] | 0.02 [0.02 - 0.03] |
| **10** | 0.17 [0.16 - 0.19] | 0.10 [0.09 - 0.12] | 0.08 [0.07 - 0.09] | 0.04 [0.04 - 0.05] | 0.08 [0.07 - 0.09] | 0.05 [0.04 - 0.05] | 0.04 [0.03 - 0.05] | 0.02 [0.02 - 0.03] |

## Supplementary Table 3. Cox cause-specific hazard models for fracture risk by site.

**ADT+ Cohort versus ADT- cohort**

| **All fractures** | **Reference** | **Adjusted HR** | **95%CI** | ***p* value** |
| --- | --- | --- | --- | --- |
| ADT (any) | None | 1.63 | [1.52; 1.75] | <0.001 |
| Age at treatment initiation | By year | 1.09 | [1.09; 1.10] | <0.001 |
| Dementia during pre-treatment period | Absent | 1.76 | [1.53; 2.02] | <0.001 |
| Diabetes during pre-treatment period | Absent | 1.13 | [1.05; 1.21] | <0.001 |
| Chronic kidney disease during pre-treatment period | Absent | 1.12 | [0.97; 1.29] | 0.133 |
| Sleep disorder during pre-treatment period | Absent | 1.31 | [1.21 1.42] | <0.001 |
| Rheumatoid arthritis during pre-treatment period | Absent | 1.53 | [1.20; 1.95] | <0.001 |
| **Vertebral fractures** | **Reference** | **Adjusted HR** | **95%CI** | ***p* value** |
| ADT (any) | None | 1.75 | [1.57; 1.95] | <0.001 |
| Age at treatment initiation | By year | 1.11 | [1.10; 1.11] | <0.001 |
| Dementia during pre-treatment period | Absent | 1.54 | [1.24; 1.92] | <0.001 |
| Diabetes during pre-treatment period | Absent | 0.98 | [0.88; 1.10] | 0.729 |
| Chronic kidney disease during pre-treatment period | Absent | 0.78 | [0.61; 1.01] | 0.056 |
| Sleep disorder during pre-treatment period | Absent | 1.36 | [1.20; 1.55] | <0.001 |
| Rheumatoid arthritis during pre-treatment period | Absent | 1.44 | [0.99; 2.11] | 0.060 |
| **Non-vertebral fractures** | **Reference** | **Adjusted HR** | **95%CI** | ***p* value** |
| ADT (any) | None | 1.65 | [1.48; 1.84] | <0.001 |
| Age at treatment initiation | By year | 1.06 | [1.06; 1.07] | <0.001 |
| Dementia during pre-treatment period | Absent | 1.32 | [1.01; 1.71] | 0.039 |
| Diabetes during pre-treatment period | Absent | 1.30 | [1.17; 1.45] | <0.001 |
| Chronic kidney disease during pre-treatment period | Absent | 1.18 | [0.95; 1.47] | 0.137 |
| Sleep disorder during pre-treatment period | Absent | 1.37 | [1.20; 1.55] | <0.001 |
| Rheumatoid arthritis during pre-treatment period | Absent | 1.42 | [0.97; 2.08] | 0.071 |
| **Hip fractures** | **Reference** | **Adjusted HR** | **95%CI** | ***p* value** |
| ADT (any) | None | 1.55 | [1.32; 1.82] | <0.001 |
| Age at treatment initiation | By year | 1.14 | [1.13; 1.16] | <0.001 |
| Dementia during pre-treatment period | Absent | 2.67 | [2.09; 3.40] | <0.001 |
| Diabetes during pre-treatment period | Absent | 1.15 | [0.97; 1.35] | 0.106 |
| Chronic kidney disease during pre-treatment period | Absent | 1.44 | [1.09; 1.89] | 0.009 |
| Sleep disorder during pre-treatment period | Absent | 1.10 | [0.90; 1.33] | 0.350 |
| Rheumatoid arthritis during pre-treatment period | Absent | 1.28 | [0.70; 2.32] | 0.424 |

## Supplementary Figure. Study design.
